# Mapping of *trans*-acting regulatory factors from microarray data

**DOI:** 10.1186/1753-6561-1-s1-s155

**Published:** 2007-12-18

**Authors:** Jeanette N McClintick, Yunlong Liu, Howard J Edenberg

**Affiliations:** 1 Department of Biochemistry and Molecular Biology, Indiana University School of Medicine, 635 Barnhill Drive, MS4063, Indianapolis, Indiana 46202, USA; 2 Department of Medicine, Indiana University School of Medicine, 714 North Senate Avenue, EF250G, Indianapolis, Indiana 46202, USA; 3 Center for Medical Genomics at Indiana University School of Medicine, 635 Barnhill Drive, MS4063, Indianapolis, Indiana 46202, USA

## Abstract

To explore the mapping of factors regulating gene expression, we have carried out linkage studies using expression data from individual transcripts (from Affymetrix microarrays; Genetic Analysis Workshop 15 Problem 1) and composite data on correlated groups of transcripts. Quality measures for the arrays were used to remove outliers, and arrays with sex mismatches were also removed. Data likely to represent noise were removed by setting a minimum threshold of present calls among the non-redundant set of 190 arrays. SOLAR was used for genetic analysis, with MAS5 signal as the measure of expression. Probe sets with larger CVs generated more linkages (LOD > 2.0). While *trans *linkages predominated, linkages with the largest LOD scores (>4) were mostly *cis*. Hierarchical clustering was used to generate correlated groups of genes. We tested four composite measures of expression for the clusters. The average signal, average normalized signal, and the first principal component of the data behaved similarly; in 8/19 clusters tested, the composite measures linked to a region to which some individual probe sets within the cluster also linked. The second principal component only produced one linkage with LOD > 2. One cluster based upon chromosomal location, containing histone genes, linked to two *trans *regions. This work demonstrates that composite measures for genes with correlated expression can be used to identify loci that affect multiple co-expressed genes.

## Background

There is a genetic component to the differences between individuals in gene expression. The confluence of techniques that allow genome-wide measurements of gene expression and the technology to examine genomic variations, single-nucleotide polymorphisms (SNPs), on a large scale allows one to map the genetic determinants of differences in gene expression. Problem 1 in Genetic Analysis Workshop 15 (GAW15) provides expression data for approximately 8800 genes, along with SNP genotypes at 2883 sites-sufficient for linkage mapping but too low a density for genome-wide association studies.

We have examined several parameters and strategies that could be used to localize regulatory elements from such data. The initial step was to check the quality of the array data and remove outlier arrays and arrays in which the gene expression did not match the gender indicated in the pedigree. We also removed genes that were not reliably detected and thereby reduced the amount of multiple testing. We are particularly interested in detecting *trans*-acting loci that regulate correlated groups of genes, because such loci should be master regulatory elements integrating expression of many genes, and have tested several strategies for detecting them.

## Methods

### Data

MAS5 signals, detection calls, and quality control (QC) information were generated from the 267 Affymetrix HG focus array CEL files (Affymetrix feature intensity files) in the GAW15 Problem 1 using R/Bioconductor [1]. The arrays were scaled to a user-specified value of 1000. Detection calls are based on a nonparametric test of the relative intensity of hybridization to the perfect match probes vs. the mismatch probes, and were calculated using the Affymetrix default parameters.

### Quality control

Arrays having either a scaling factor or percent present with values outside of the median ± 3 times the inter-quartile range were eliminated (1341_12_rep1, 1362_01_rep1, 1362_01_rep2, 1416_02_rep1, 1418_02_rep1, 1423_13_rep2, 1424_01_rep2). We identified genes with sexually dimorphic expression by comparing (using Welch's *t*-test) the 54 arrays from men with the 51 arrays from women in the grandparents generation. Among duplicate arrays we selected the one with QC values nearest to the median.

### Selection of probe sets and generation of clusters

Coefficient of variation (CV: standard deviation/mean) for each probe set was calculated. One hundred probe sets were randomly selected from each of three groups: CV between 0.65 and 0.80, CV between 0.40 and 0.45, and random.

Hierarchical clustering (using correlation coefficient as the distance measure, and complete linkage) was carried out in Matlab (version 7.2, Mathworks) to generate groups of probe sets that have similar expression patterns. Thirty-three clusters were generated with a minimum correlation coefficient ≥ 0.60 and containing at least six probe sets. Composite measures of expression for each cluster were generated from 1) the mean of the signals, 2) mean of normalized signals ([signal-mean]/SD), and 3) projections of each array on the first two principal components of the normalized gene expression signals. The latter measurement indicates the expression levels of the first two eigengenes on each array; singular value decomposition (SVD) was conducted to calculate the eigengene and eigenarray matrices using the normalized signal [2].

We also clustered co-expressed genes that were located nearby on a chromosome. The probe sets were mapped onto chromosomes; all the probe sets within 2 Mb downstream of a target probe set were considered neighbors. A co-expressed neighbor was defined as a neighboring probe set that had a similar expression pattern as the target probe set (correlation coefficient > 0.4). For each probe set, the probability that observing ≥*n *co-expressed neighbors, by chance, in a neighborhood with *N *neighboring probe sets was calculated based on the binomial distribution. The false-discovery rate (FDR) of the significant co-expressed neighboring clusters was calculated [3].

### Linkage

Linkage analysis was performed using SOLAR [4]. The map file was created using the Rutgers map data gathered by Sung et al. [5] and the SNP data from 193 individuals. Genotypes were removed if they did not follow Mendelian patterns of inheritance. Multipoint analysis was performed on the MAS5 signals using the *tdist *option which uses a robust estimation of mean and variance that can adjust for excess kurtosis. Given the resolution of the linkage map, we considered linkage to a region within 10 Mb of a gene to be *cis*, and more distant linkages *trans*.

## Results

### Quality control issues

We first examined quality control data and removed arrays that were outliers. Comparing male and female founders in the GAW Problem 1, we detected three probe sets with robust sex specific expression: female: 214218_s_at (*XIST*); male: 205000_at and 206700_s_at (both on Y chromosome). Five arrays with sex-specific expression inappropriate for the pedigree information were removed (1418_08_rep1, 1418_14_rep1, 1423_12_rep2, 1423_13_rep2, 1423_14_rep2). The QC evaluation and the removal of duplicates left 193 people in the pedigrees, 190 of whom had expression data. For the three remaining (1362_1, 1424_1 and 1418_14) only genotype information was used.

**Figure 1:**
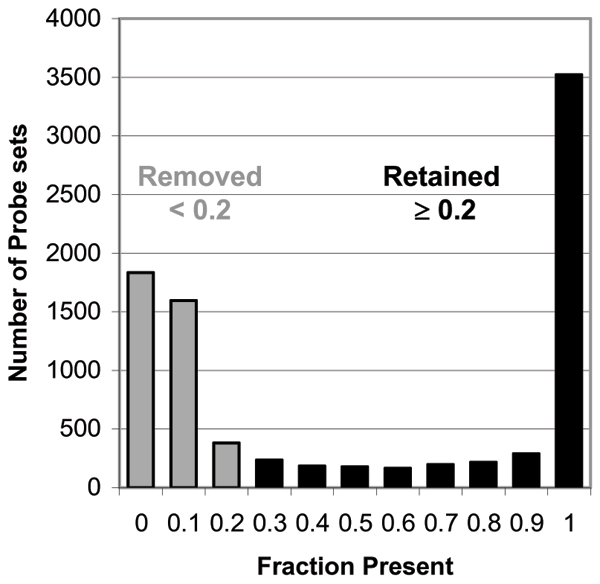
Distribution of fraction present for all probe sets on the arrays.

Control probe sets and those measuring transcripts which are spiked (44 probe sets) were removed, leaving 8749. The distribution of present calls is shown in Figure 1. To avoid analyses of genes that were not detectably expressed (and therefore represent noise), probe sets that were called present on fewer than 20% of the 190 arrays were removed from the analyses [6]; 3757 probe sets were removed, leaving 4992.

### Selection by CV

**Figure 2:**
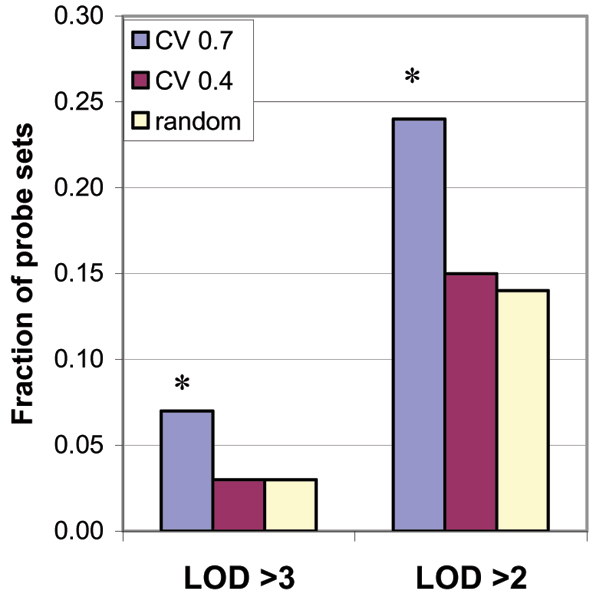
Fraction of probe sets with large LOD scores for each group of selected probe sets.

For the 300 probe sets selected to test the effects of CV, there were 13 linkages with a LOD score ≥ 3.0 (Table 1) and 40 with LOD between 2 and 3. The group of probe sets with higher CV produced a larger number of significant (LOD > 3) and suggestive (LOD > 2) results (Figure 2, Table 1). Given the limited numbers of probe sets analyzed, the differences between the groups were only suggestive (*p *= 0.1, for LOD > 3 and LOD > 2). Most of the linkage results (44/53) with LOD ≥ 2 were *trans*, but the larger LOD scores were more likely to be *cis*; five of seven with LOD ≥ 4 were *cis *(Table 1). One probe set (205469_s_at) had both a *cis *and *trans *linkage with LOD = 2.1 and 2.2, respectively.

**Table 1:** Probe sets with LOD > 3.0

Probe set^a^	LOD	Chr^b^	cM^c^	Linkage	Gene location	ENTREZ gene	UniGene ID	Gene symbol	Group^d^
65588_at	9.27	20	62	*cis*	chr20q11.23	388796	Hs.400876	LOC388796	CV40
219759_at	7.6	5	109	*cis*	chr5q15	64167	Hs.482910	LRAP	CV70
320_at	5.12	6	59	*cis*	chr6p21.1	5190	Hs.567243	PEX6	CV40
212509_s_at	4.49	17	113	*cis*	chr17q25.1	439921	Hs.250723	MXRA7	CV70
205018_s_at	4.49	21	20	*trans*	chr13q32.1	10150	Hs.125715	MBNL2	CV70
204418_x_at	4.25	1	145	*cis*	chr1p13.3	2946	Hs.279837	GSTM2	CV40
205027_s_at	4.03	9	98	*trans*	chr10p11.23	1326	Hs.432453	MAP3K8	CV70
203868_s_at	3.79	21	30	*trans*	chr1p32-p31	7412	Hs.109225	VCAM1	CV70
204073_s_at	3.77	11	56	*trans*	chr11q12-q13.1	745	Hs.473109	C11orf9	CV70
208121_s_at	3.57	9	97	*trans*	chr12p13-p12	5800	Hs.160871	PTPRO	CV70
211317_s_at	3.44	8	74	*trans*	chr2q33-q34	8837	Hs.390736	CFLAR	random
204015_s_at	3.29	12	122	*trans*	chr8p12-p11	1846	Hs.417962	DUSP4	random
204908_s_at	3.23	21	29	*trans*	chr19q13.1-q13.2	602	Hs.31210	BCL3	random

^a^Probe sets among the 300 selected to test selection by CV. Annotations were from Netaffx, .

^b^Chromosome of linkage peak

^c^Centimorgan distance of linkage peak

^d^Group indicates which CV group this probe set was in (see Methods).

### Clusters of genes with correlated expression

**Figure 3:**
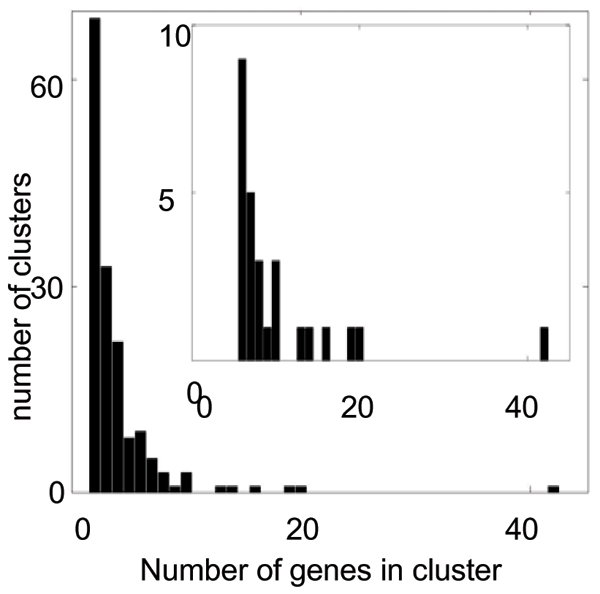
Characterization of the clusters.

We defined 33 gene clusters by cutting off the hierarchical tree at a minimum correlation coefficient between two branches of 0.6. We focused on the 26 clusters that had an average correlation > 0.7 and contained at least six genes (Figure 3). Initial analyses showed that clusters with CV < 0.3 gave nearly no linkages with LOD score > 2 (1 of 89 probe sets in the first six such clusters) so we did not analyze the remaining clusters with CV < 0.3.

In the 19 clusters used for linkage, there were 28 individual probe sets that had LOD scores > 2.0. All 28 linkages were *trans*. Ten of the 19 clusters had at least one probe set or composite measure with LOD > 2. Eight of these ten contained multiple probe sets or composite scores that linked to the same chromosomal region. Three clusters had multiple regions with more than one linkage to them. In all eight clusters, the composite measures linked to one of the multiply-linked regions. In most (seven of eight clusters), the individual probe set with the largest LOD score exceeded the LOD score achieved by the composite measures that linked to the same region. Among the composite measures, the first principal component and the two mean signals (raw and normalized) all linked to the same chromosomal region with very similar LOD scores. The first principal component had an average relative variance (proportion of variance captured) of 0.41 (range, 0.26 to 0.53, Table 2.) The first PC relative variance was larger in clusters with fewer probe sets. The second principal component generally produced poor results: LOD < 1.3 for most, only one cluster with LOD > 2.

**Table 2:** Characteristics of clusters used for linkage analysis

Probe sets^a^	Average correlation^b^	PC1 content^c^	Mean CV^d^	PC1 LOD^e^	Average LOD^f^	Highest probe set LOD^g^	Multi-linked regions^h^
6	0.83	0.53	0.56	2.2	2.5	2.4	1
42	0.82	0.29	0.20	-^i^	-	0.9	0
10	0.79	0.42	0.17	-	-	0.8	0
6	0.77	0.49	0.44	2.4	2.4	3.4	2
19	0.77	0.32	0.46	2.6	2.6	3.6	4
20	0.76	0.31	0.37	-	-	1.6	0
6	0.75	0.47	0.43	3.0	3.0	3.4	1
7	0.75	0.45	0.41	1.1	1.4	1.6	0
10	0.75	0.39	0.52	2.3	2.9	3.2	1
9	0.74	0.40	0.39	0.3	0.8	0.9	0
6	0.74	0.46	0.32	0.4	0.4	0.7	0
8	0.74	0.41	0.47	2.9	2.9	4.0	3
6	0.73	0.46	0.39	1.8	2.1	1.9	0
8	0.73	0.41	0.29	1.5	1.6	1.9	0
6	0.73	0.45	0.29	2.1	2.4	2.4	0
7	0.73	0.43	0.37	1.4	1.5	1.9	0
16	0.72	0.31	0.34	1.6	1.7	2.4	1
13	0.72	0.33	0.28	-	0.8	1.6	0
10	0.71	0.36	0.27	-	1.4	2.9	1

^a^Number of probe sets within the cluster

^b^The average correlation between probe sets in the cluster

^c^The amount of variation captured by the first principal component

^d^Mean CV for all probe sets in the cluster

^e^LOD scores for first PC

^f^Highest LOD score for composite generated from average signal or average normalized signal

^g^Highest LOD score for an individual probe set

^h^The number of chromosomal regions linked to by multiple probe sets or composite measures with at least one with LOD > 2

^i^- indicates no linkage

Two of the 19 clusters analyzed contained ribosomal proteins, with correlation near 0.8 and a CV ≤ 0.2. In these two clusters there were no LOD scores > 2, but many of the probe sets and the composite measures linked to chromosome 3 at 188 to 193 cM at lower LOD scores (Table 2).

### Clusters of co-expressed neighboring genes

There were six chromosomal regions containing significant clusters of co-expressed genes (at FDR < 5%). We focused on the two regions that contained more than 10 co-expressed neighboring genes. No chromosomal region linked to the cluster on 11q13.1 neighboring probe set 204441_s_at. The cluster on 6p21.3, starting from probe set 209398_at, had an average correlation coefficient of 0.50. Interestingly, all the 11 co-expressed genes in this 6p21.3 cluster were histone genes. The first principal component contained 55.7% of the variance, and linked to chromosome 5 at 144 to 145 cM at rs880080 (LOD = 2.6). There were 226 annotated genes located within a 14-Mb (1 LOD) region. Gene ontology analysis indicates that 17 of the 226 genes related to transcriptional regulation and 6 related to the cell cycle. These factors include bromodomain containing 8, taf7, RNA polymerase II TATA box binding protein (TBP)-associated factor, histone deacetylase 3, glucocorticoid receptor, and transcription elongation regulator 1. The second principal component contained 13.1% of the variance, and linked to chromosome 21 at 29 cM (LOD = 1.5). Seven out of 119 genes that fell in the linkage region were transcription factors, and one was related to cell cycle.

## Discussion

Pre-cleaning the data to remove outlier arrays or arrays with other problems (e.g., expression data inconsistent with nominal gender) is important, but not always done. Beyond that, we have found that removing all data from probe sets not reliably detected in at least a reasonable fraction of the arrays removes noise, reduces multiple comparisons, and improves the ability to detect real differences [6]. We used a fraction present of 0.20 as the cut-off based on the distribution of this measure in the present dataset (Figure 1).

A minimum amount of variation in expression appears to be required to detect linkage. Probe sets or groups with a CV < 0.30 did not yield many LOD scores > 2.0. We found a trend: probe sets with larger variation (larger CV) produced more significant or suggestive LOD scores (Figure 2).

*Trans *linkages predominated, not just for the clusters but also for 300 individual probe sets used for the CV comparison: 45 of 53 (85%) of the linkages with LOD > 2 were *trans*. Seven were *cis *(13%; 5 were within 5 Mb) and one gave both a *cis *and *trans *linkage. Morley et al. [7] also found skewed results, with 77.5% of linkages being *trans*, 19% *cis*, and 3.5% with two or more linkages. Part of the explanation for the excess of *trans *linkages may be the multiple comparisons: for a *cis*-linkage, only a limited number of SNPs in the region of the gene are relevant, whereas for a *trans*-linkage all probe sets are tested against each expression value. Thus, many *trans*-linkages may represent false positives due to a higher degree of multiple testing.

Despite the fact that most linkage results with LOD ≥ 2 were *trans*, the larger LOD scores were more likely to be *cis *(5 of 7 with LOD ≥ 4.0). A likely explanation of this skewing of results is that multiple *trans *QTLs may each have small effects on gene expression, while *cis *effects may be much stronger. Transcriptional regulation involves the binding of multiple *trans*-acting transcription factors to the regulatory region (*cis*-acting elements) of a given gene. Thus, the *cis*-acting elements of a gene, located in reasonable proximity to it, integrate the effects of multiple *trans*-acting transcription factors.

Three of the four composite measures used for the clusters (first principal component, mean of raw signal, and mean of normalized signal for all probe sets in the cluster) gave similar results. They all linked to the same region when the LOD score was >2.0, and usually when it was >1.0. In most cases the linkage resulted in similar LOD scores. The first principal component was less likely than the average expression levels to have a normal distribution and was more difficult to transform to a normal distribution, suggesting that the mean signal (or normalized signal) is a better measure to use for these analyses and eliminating the need for SVD analysis. The composite scores did not produce stronger linkages to *trans*-acting loci than individual probe sets. However, they may be useful to identify those loci that affect multiple correlated genes.

We compared a cluster of histone genes generated based on genes with correlated expression (six probe sets, first row in Table 2) with a cluster based on location along the chromosome (correlated neighboring genes, 11 probe sets). Three probe sets were common to both clusters. The composite scores from both clusters performed very similarly, with LOD scores ranging from 2.2 to 2.6 and all linking to the same region. The average normalized signal of the cluster of neighboring genes produced the largest LOD score, which was larger than any individual probe set that linked to the same region from either group. The linkage was to regions containing many transcription factors and cell cycle-related genes, which makes biological sense.

## Competing interests

The author(s) declare that they have no competing interests.
